# HIS-based Kaplan-Meier plots - a single source approach for documenting and reusing routine survival information

**DOI:** 10.1186/1472-6947-11-11

**Published:** 2011-02-16

**Authors:** Bernhard Breil, Axel Semjonow, Carsten Müller-Tidow, Fleur Fritz, Martin Dugas

**Affiliations:** 1 Department of Medical Informatics, University Muenster, Domagkstraße 9, 48149 Münster, Germany; 2 Department of Urology, Prostate Centre, University Hospital Muenster, Albert-Schweitzer-Straße 33, 48149 Münster, Germany; 3 Department of Medicine A, Haematology and Oncology, University Hospital Muenster, Domagkstraße 3, 48149 Münster, Germany

## Abstract

**Background:**

Survival or outcome information is important for clinical routine as well as for clinical research and should be collected completely, timely and precisely. This information is relevant for multiple usages including quality control, clinical trials, observational studies and epidemiological registries. However, the local hospital information system (HIS) does not support this documentation and therefore this data has to generated by paper based or spreadsheet methods which can result in redundantly documented data. Therefore we investigated, whether integrating the follow-up documentation of different departments in the HIS and reusing it for survival analysis can enable the physician to obtain survival curves in a timely manner and to avoid redundant documentation.

**Methods:**

We analysed the current follow-up process of oncological patients in two departments (urology, haematology) with respect to different documentation forms. We developed a concept for comprehensive survival documentation based on a generic data model and implemented a follow-up form within the HIS of the University Hospital Muenster which is suitable for a secondary use of these data. We designed a query to extract the relevant data from the HIS and implemented Kaplan-Meier plots based on these data. To re-use this data sufficient data quality is needed. We measured completeness of forms with respect to all tumour cases in the clinic and completeness of documented items per form as incomplete information can bias results of the survival analysis.

**Results:**

Based on the form analysis we discovered differences and concordances between both departments. We identified 52 attributes from which 13 were common (e.g. procedures and diagnosis dates) and were used for the generic data model. The electronic follow-up form was integrated in the clinical workflow. Survival data was also retrospectively entered in order to perform survival and quality analyses on a comprehensive data set. Physicians are now able to generate timely Kaplan-Meier plots on current data. We analysed 1029 follow-up forms of 965 patients with survival information between 1992 and 2010. Completeness of forms was 60.2%, completeness of items ranges between 94.3% and 98.5%. Median overall survival time was 16.4 years; median event-free survival time was 7.7 years.

**Conclusion:**

It is feasible to integrate survival information into routine HIS documentation such that Kaplan-Meier plots can be generated directly and in a timely manner.

## Background

Accurate survival or outcome information is important for many clinical studies, clinical routine and epidemiology. The standard method for estimating survival is the Kaplan-Meier plot (KM-plot) [1] with high relevance in medical research. Particularly in oncology the Kaplan-Meier technique is used to compare survival information between different therapy strategies or stages of the disease [2]. Survival time and follow-up status are used to compute an estimate of a survival curve for censored data.

In the German healthcare system a distinction can be made between the routine documentation which is commonly performed in hospital information systems or still paper based and the research documentation mainly performed in electronic data capture systems (EDC) or on paper based case report forms (CRF). There are several systems in healthcare resulting in separate documentation of medical routine, research and quality management data. In the context of clinical studies survival information is currently captured on paper based CRF and occasionally on electronic CRF (eCRF), but generally separated from HIS. However, some patients already have at least a basic electronic medical record [3]. Currently survival analysis is not possible within the HIS as the required data is not available. A problem of these external databases consists in the resulting difficult multiple usage of information. Data from a single patient can be relevant for several studies (e.g. therapy study, biomarker discovery study, epidemiological studies) in addition to clinical routine. Redundant documentation is common but inefficient regarding a resource limited setting and carries the danger of inconsistent information. In this setting it would be attractive to use health data outside of direct care delivery. A secondary use of documented data for research and quality management [4] may be of potential benefit for those physicians who already use electronic documentation [5]. For instance, the idea of the REUSE project implies that clinical data should be based in the electronic health record (EHR) independent from the context, in which data is captured [6]. The secondary use of clinical data has enormous potential for improving quality of care [7,8].

Furthermore, there is heterogeneity in the required follow-up documentation depending on the disease and the department in which the data is obtained. Physicians need further information to interpret the status in combination with clinical data. Also, the parameter values for the follow-up status may be different between oncological diseases. Therefore, an efficient implementation should be based on a generic data model which is suitable for several diseases.

Studies of health services research, epidemiological studies and phase III/IV studies often consist of a high number of cases resulting in laborious documentation and high data management costs. Using routine documentation may reduce the documentation workload and reduce costs. To reach these goals the respective source data have to be "accurate, legible, contemporaneous, original, attributable, complete and consistent" [9].

However, data quality of follow-up documentation is often unsatisfactory [10] and requires adaption before it can be used for research. The follow-up documentation needs to be complete to obtain meaningful KM-plots as incomplete information can bias the analysis of the results [11]. One strategy to increase completeness and achieve higher data quality is to use an electronic documentation tool [12,13]. This approach could be extended to use the HIS for clinical and research documentation which would also result in high data quality [14]. Commercial HIS usually cover only events during hospitalisation so there is a need for a special follow-up module to allow the survival documentation for inpatients and outpatients.

With respect to clinical quality management it is important to obtain timely KM-plots of all patients and not just from those in clinical studies. Thus, it would be desirable to integrate the follow-up documentation into clinical routine and document it in the EHR within the HIS. Using this method, it will be made available for all treating physicians and clinical research projects can be combined with routine documentation by reusing the EHR [15].

Because of the high relevance, especially in oncology, we analyse whether an integrated follow-up documentation is feasible and focus on the following objectives:

1. Is it feasible to design a follow-up documentation system in the HIS which is suitable for several oncological diseases and provides a secondary use of data?

2. Is it possible to extract survival information from routine HIS documentation so that physicians can obtain KM-plots in a timely manner?

3. What level of data quality can be achieved with respect to completeness of forms and completeness of items per form?

## Methods

### Process analysis

We analysed the current follow-up documentation process in the urology and haematology department at the University Hospital Muenster. Physicians were interviewed to identify weak points and requirements during the follow-up appointments.

### Form analysis

During the form analysis we investigated the attributes which characterise the overall survival (OS) and the event-free survival (EFS). We compared follow-up documentation of two tumours regarding type, complexity and concordances.

### Concept, data model and implementation

We developed a concept for comprehensive survival documentation based on a generic data model which is described in the CDISC Operational Data Model (ODM v1.3.1.) [16] using SNOMED CT V3 Codes [17]. Based on this concept an electronic survival form was implemented within the local HIS containing specialised parts for leukaemia and for prostate cancer. We used the integrated tools of the HIS (ORBIS^® ^from Agfa Healthcare) [18] to create a form which contains all relevant survival data for KM-plots and integrated it into the clinical workflow.

### Data export and survival analysis

We created a report of the HIS form to extract follow-up information (initial diagnosis date, initial therapy date, date of last contact, status at the last contact, etc.) from the HIS and to transfer pseudonymized data sets to statistical programs. This report was integrated in the HIS in such a way that it can be used by the physicians to view summarized data of their patients in the HIS or to export pseudonymized survival data as comma separated values (CSV). Survival analyses were implemented in R (version 2.10.1) and for the KM-plots we used the "*survfit function*" of the R survival library [19]. Differences between subgroups of patients were assessed using log-rank test, implemented in the "*survdiff function*". A batch script was written to execute the R survival function in such a way that survival curves and data quality information are directly accessible in a PDF-file.

### Analysing data quality

We analysed completeness to describe data quality according to Chan et al [20]. Therefore, we designed a report to query missing and incomplete forms. Based on these export data, completeness of forms (reference: all prostate cancer patients in urology) and completeness of documented items per form were analysed.

The study was performed in compliance with the World Medical Association Declaration of Helsinki on Ethical Principles for Medical Research Involving Human Subjects. HIS data access was approved by the responsible data protection officer, only de-identified data items were exported.

## Results

### Process analysis of the paper based follow-up documentation

Follow-up documentation was paper based for AML patients in haematology and spreadsheet based for prostate cancer patients in urology. In both departments, forms were completed when patients were appointed to follow-up examinations. If the patient lacked a follow-up date the treating physicians could obtain the information from general practitioners, from the epidemiology cancer registry or from the registration office (in case of fatality). For statistical analysis and survival curves the follow-up documentation was entered manually into spreadsheets or statistical programs which were then used to generate KM-plots.

### Form analysis

Each department has its own follow-up form. While analysing both forms we identified 13 common attributes. Particularly the relevant survival information (diagnosis date, therapy date, follow-up status and follow-up date) are common to both departments so that a generic form for both diseases is possible. The remaining attributes of both departments were not included in the generic data model but belong to the disease specific documentation which is considered in a specialised part of the form. Table 1 shows the results of the form analysis.

**Table 1:** Results of the form analysis

*Department*	*#Pages*	*#Attributes (total)*	*#Attributes (common)*
Urology	1	35	13

Haematology	1	30	13

Follow-up forms from both departments contain more than 30 attributes to document survival information, 13 of which are common (e.g. diagnosis date, therapy date, follow-up date, follow-up status).

Two of the common attributes (study, status) have different parameter values. For example, the values of the status lists differed between the two departments considering the different stages of the diseases. Therefore we implemented a catalogue with variable parameter values for each department. For the KM-plots it is only relevant to distinguish between overall survival (OS --> yes/no) and event free survival (EFS --> yes/no). Therefore, we mapped the more detailed elements of the status list unambiguously to these two parameters. The resulting status list with the mapping and coding is presented in table 2. It is now possible to use this encoding in both departments for Kaplan-Meier plots.

**Table 2:** Status values in the department of haematology and urology

*Status in the haematology department (AML)*	*Status in the urology department (prostate cancer)*	*OS*	*EFS*
Initial diagnosis	Initial diagnosis	0	0

-	Relapse free	0	0

Aplasia	-	0	0

First remission	-	0	0

Complete remission unconfirmed	-	0	0

Second remission	-	0	0

Relapse	Relapse (PSA)	0	1

-	Relapse (imaging)	0	1

Persistent AML	-	0	1

Death of AML	Death of prostate cancer	1	1

Death independent from AML	Death independent from prostate cancer	1	1

Death of unknown cause	Death of unknown cause	1	1

Both departments have different status list values to be considered in the survival analysis. Concerning overall survival (OS) analysis all death events are coded as 1, regarding event-free survival (EFS) analysis all except for initial diagnosis and relapse free are coded as 1.

### Concept and data model

**Figure 1:**
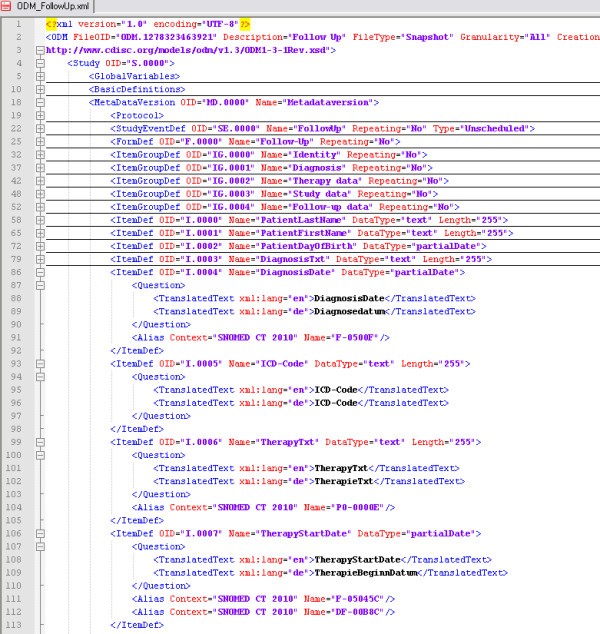
**Extract of the follow-up representation in CDISC ODM**. The extract shows the definition of diagnosis and therapy data with their data type. The complete ODM example of the follow-up form can be found in the supplement (see Additional file 1).

Based on the results of the form analysis we developed a generic data model which includes the 13 common attributes. These attributes were determined through data types and also tagged with SNOMED CT codes. For the specification of the data model we used CDISC ODM because it is designed to facilitate interchange of metadata and data for clinical research [16]. The ODM follow-up form consists of 5 item groups (identity, diagnosis, therapy, study data and follow-up data). An extract of this form is shown in figure 1. The complete ODM example of the follow-up form can be found in the supplement (see Additional file 1). All items are specified by name, id and data type. For all attributes we added SNOMED CT codes so that the used concepts are well-defined. Regarding the different status values and the different study lists in each department we modelled code tables for the parameter values of the follow-up status. Finally we obtained a system independent specification for the follow-up form, which also allows semantic interoperability through the SNOMED codes.

### Implementation

**Figure 2:**
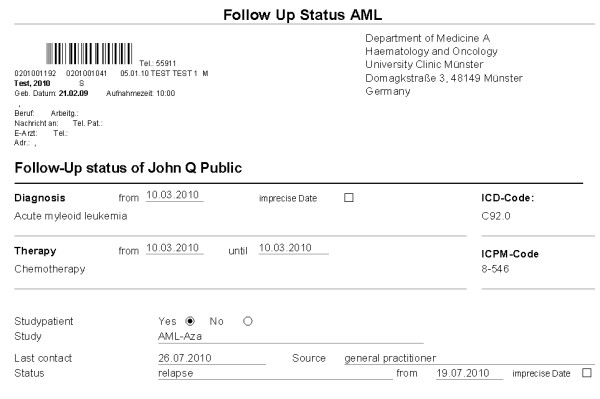
**Extract of the follow-up form**. Initial diagnosis and initial therapy can be documented with date, text and classification in the upper part of the form. The lower part contains the date of the last visit with the associated status in addition to the studies. This figure shows the electronic version of the AML follow-up documentation. All attributes are similar for prostate cancer but status lists and studies have different parameter values. In addition to this generic form, disease specific form components can be provided.

Based on the generic data model we implemented a follow-up form within our HIS (ORBIS^®^) and introduced the electronic follow-up documentation for prostate cancer patients in the urology department and for AML patients in the haematology department. An extract of the implemented HIS form, used for routine documentation, is shown in figure 2. For each patient the initial diagnosis and initial therapy can be specified with date, text and classification. Hence, both points of time can be used as start date of the KM-plots. The lower part of the form contains the date of the last visit with the associated status and provides the possibility to document the source of the survival information (e.g. general practitioner, registration office). In addition the participation in clinical studies can be documented. We also added disease specific sub-forms for prostate cancer and AML containing the attributes which were not common in both departments. These forms complete the follow-up documentation in both departments.

In routine documentation the follow-up form should be completed for every cancer patient in the respective department. Patients with prostate cancer or leukaemia have regular follow-up examination and during those visits the survival information is documented. If patients do not appear the information has to be collected from the current practitioner. In this case physicians and study nurses enquire relevant information from general practitioners as well as the registration office (if there is a fatality reported) and enter this information into the system. We also added the attribute "source" so that the documenting physician can document the enquired institution. In this manner information from outpatients can be also documented. In addition to this generic form, disease and department specific attributes can be documented in specific form components which were provided for prostate cancer and leukaemia. To analyse the feasibility of KM-plots based on structured HIS data the form was integrated in the clinical workflow in the urology department and during a short time frame data from follow-up patients was documented. To achieve a high number of cases which allow for comprehensive analyses and to reuse previously existing survival data from paper based records or spreadsheet files in the urology department this information was retrospectively transferred to the HIS. In the haematology department up until now the electronic follow-up documentation was only used in a pilot installation with relatively few patients. Therefore, the following analyses are based on urology data. In our approach we provide one form per follow-up date so that each patient has several forms during the course of his disease. The current survival status is always taken from the most current form.

### HIS-based Kaplan-Meier

The documented survival data were extracted from the EHR and analysed with the statistic software R. We considered a follow-up period from 03.06.1992 to 31.05.2010 in which patients where documented and completed follow-up forms also retrospectively to obtain a relevant data basis for the KM-plots. In total follow-up forms were entered from 23^rd ^February to 01^st ^July 2010 for 1029 patients; 223 of them were completed in the routine documentation process, 806 were entered retrospectively. Using the R survival library we implemented survival analyses and KM-plots based on exported CSV-data. First we removed all duplicated forms from all patients and kept only the data from the most current form. Observation time was defined on basis of therapy start date and follow-up date, overall survival and event-free survival were computed concerning the following status encodings (table 2). We created data frames for the EFS and OS for the patient collective as well as data frames for grouped analyses. Prostate cancer patients were divided into two groups. KM-plots with numbers at risk were generated from all four data frames. The complete R code can be found in the supplement (see Additional file 2).

Survival information was available for 881 of the 965 patients. The median overall survival time was 16.4 years, the median event-free survival time was 7.7 years. The probability of 5-year overall survival was 98.2% (EFS: 82.8%), the probability of a 10-year overall survival was 89.9% (EFS: 32.6%). Table 3 shows the basic information of patients in the urology department.

**Table 3:** Survival information of patients in the urology department

*Department of urology*	*Analysis of overall survival*	*Analysis of event-free survival*
Number of patients	965	965

Survival information available	881	881

Median survival time (years)	16.4	7.7

Probability of 5-year survival (percent)	98.2	82.8

Probability of 10-year survival (percent)	89.9	32.6

Total number of events	31	267

->Death	31	31

->Relapse	-	236

Survival information was available for 881 patients covering a time interval of nearly 17 years.

**Figure 3:**
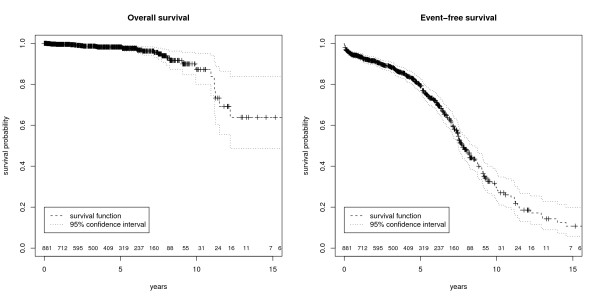
**Kaplan-Meier Plots**. These figures show the survival with 95% confidence interval and numbers at risk. The left figure represents overall survival and the right figure event-free survival of patients with prostate cancer after radical prostatectomy.

**Figure 4:**
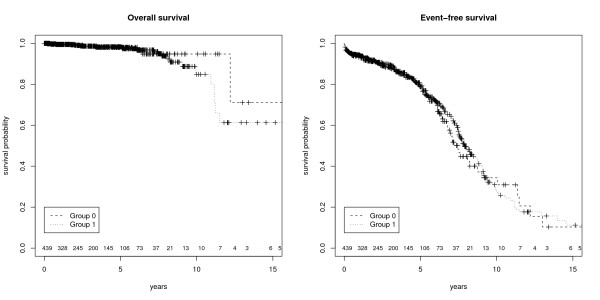
**Kaplan-Meier Plots (Group analysis)**. These figures show the survival of the prostate cancer patients divided into two groups with numbers at risk. The left figure represents overall survival with no significant difference (p = 0.552) and the right figure event-free survival which shows a significant difference between both patient groups (p < 0.001).

Based on these survival data from HIS the following KM-plots (figure 3+4) were created. Figure 3 shows the overall and the event free survival with a 95% confidence interval of all prostate cancer patients documented in the HIS. Figure 4 illustrates that is also possible to obtain group analyses. To differentiate between the patients it is possible to combine survival data with other routine information (e.g. biopsy information, lab results, body weight, height, ultra sound findings) available in the EHR.

We wrote a batch script to automate the process from the export file to the final PDF with the KM-plots. During several discussions with health professionals we discovered that there are patients for whom no accurate date of relapse or freedom of relapse can be specified. To handle this, the generic data model was adapted to allow for imprecise inputs (e.g. Oct. 2008, 2007).

### Data quality

Based on the exported data sets we assessed completeness of the documentation to describe data quality. With a focus on the mandatory items for the survival analyses we used R to identify missing items of the survival parameters used for the KM-plots (therapy start date, follow-up status and follow-up date). These reports are permanently installed so that data quality may be obtained also for future analyses and interpretation of the KM-plots. Concerning the documented items per form, we distinguished between routine cases and retrospective cases and measured also the completeness of the whole data set. During the routine documentation the completeness of all items was 86.6% due to a few missing follow-up dates. Considering only retrospectively entered data we reached a completeness of 92.4%. In total, the completeness in the follow-up date was high (~94.3%) while completeness of therapy start date and follow-up status was very high (> 97%). KM-plots based on all three attributes were available for 881 patients (91.3%). Table 4 shows the results.

**Table 4:** Completeness of survival data

*Attribute*	*Therapy start date*	*Follow-up Status*	*Follow-up date*	*All three available*
Routine cases	207			
Available	204	207	183	180
Completeness	98.5%	100%	88.4%	86.6%
Retrospective Cases	758			
Available	738	743	727	701
Completeness	97.3%	98%	95.9%	92.4%
Total Cases	965			
Available	942	950	910	881
Completeness	97.6%	98.5%	94.3%	91.3%

Completeness was measured for routine cases (207) and retrospective cases (758) separately. In total therapy date and follow-up status is relatively complete for all patients (> 97%), follow-up date was available for 94.3% of the patients. Imprecise date items (follow-up month and follow-up year available) are considered. Survival information was available for 91.3% of the patients.

To analyse the acceptance of the electronic forms we measured how many patients with prostate cancer diagnosis already have an electronic follow-up form. For the analysis of the completeness of forms, we considered patients with a main diagnosis of prostate cancer from 01.01.2009 to 31.12.2009 in the department of urology. We chose this time range because patients with recent diagnoses are currently not appointed to follow-up examination and could bias the result. In total 115 of the 191 patients have at least one follow-up form with survival information so that completeness of forms is 60.2%.

## Discussion

With our implementation it is now possible to generate KM-plots from routine data. After exporting follow-up data sets in a pseudonymized format from the HIS the physician can start the batch script with the R survival functions to obtain a resulting PDF-file with the KM-plots. Both actions can be done by physicians within a minute and therefore it is a feasible method to get timely curves from the current data. Up to now, this procedure was not possible for physicians because data from routine documentation has to be transferred into statistical programs for survival analyses to be performed. The idea to enhance the primary information system is not a new one. Previously, in 1996, Balas et al. state that "to manage care and improve quality, primary care computer systems should incorporate these effective information services" [21]. However, the implementation of single source systems as described by Kush et al. [22] is still rare. During our literature search, we failed to find similar approaches of integrating this kind of follow-up documentation in the HIS in such a way that timely survival curves can be generated. Ene-Iordache et al. analysed regulatory-compliant eCRF [23] and Embi et al. identified in 2009 a lack of tools for clinical research activities [24]. By using electronic point-of-care documentation to generate KM-plots clinical research activities can be supported and the integrated documentation contributes to the single source approach.

### Data Quality Aspects

The measured data quality, especially the completeness of documented items per form, was high but most of information was transferred from retrospective data collections (paper based documentation and spreadsheets). The completeness of the electronic forms in the HIS (retrospective and current documentation) was only 60.2% so we assume that there are still patients with missing or paper based documentation. However, to analyse the completeness of forms more data is needed. Further analyses will show the differences in completeness of forms between the retrospective collection and the routine documentation. Chan et al. reviewed data quality in EHRs of recent studies and reported that data completeness varied substantially across studies and that even in the same organisation the amount of missing data is varying [21]. To measure follow-up completeness Clark et al. introduced the ratio of the total observed person-time of follow-up as a percentage of the potential time of follow-up in a study [11]. Therefore we measured follow-up completeness of C = 76.9% using this approach for all prostate cancer patients who underwent radical prostatectomy in the urology department.

The follow-up documentation is heterogeneous and therefore the implementation in a local HIS is complex and time consuming. There is currently no possibility to reuse captured routine data in electronic study documentation systems and therefore the advantages of HIS based documentation are limited. Especially studies with a high number of study cases (e.g. epidemiological studies, phase IV studies) are attractive for data re-use. The follow-up documentation is usually scheduled in defined intervals (depending on the disease). The HIS-based approach adds the functionality to notify the treating physicians of required documentation activities. We plan to integrate work lists which show all patients without a follow-up form in the last year. This approach could be extended by an automated creation of forms in the HIS related to the follow-up intervals. Survival information in the HIS can be re-used for physician letters. Our approach of follow-up documentation is generic and we intend to extend it to other departments and diseases. If the follow-up information is available for many patients it can also be used for patient recruitment for clinical trials [25-28] as attributes like survival status are now documented in a structured way within the HIS and can be used as inclusion or exclusion criteria.

In a short time frame 223 cases were documented during routine documentation which indicates a good clinical acceptance. In addition 806 cases were completed retrospectively in order to have the entire follow-up documentation electronically available showing the need for HIS based follow-up documentation. Structured documentation of follow-up items should therefore be a standard functionality in HIS.

## Conclusion

Different follow-up forms can be united in a comprehensive module which is suitable for oncological diseases. The integration in the local HIS is feasible and provides possibilities to reuse this information for quality control and clinical research (single source) by generating timely survival curves from routine data.

## Abbreviations

AML: acute myeloid leukaemia; CDISC: Clinical Data Interchange Standards Consortium; CRF: case report form; eCRF: electronic case report form; CSV: comma separated values; EDC: electronic data capture; EFS: event-free survival; EHR: electronic health record; HIS: hospital information system; ODM: Operational data model; OS: overall survival; PDF: portable document format; PSA: prostate specific antigen; SNOMED CT: Systematized Nomenclature of Medicine - Clinical Terms;

## Competing interests

The authors declare that they have no competing interests.

## Authors' contributions

BB implemented the HIS forms and reports, created the survival curves, analysed data quality and wrote the manuscript. AS and CMT provided clinical data and survival information. FF supported implementation of forms and MD contributed the data quality analysis. FF and MD critically revised the manuscript. All authors read and approved the final manuscript.

## Pre-publication history

The pre-publication history for this paper can be accessed here:

http://www.biomedcentral.com/1472-6947/11/11/prepub

## Supplementary Material

Additional file 1 **ODM Follow-up form**. This xml-file contains the follow-up form in the ODM format (v1.3.1). Click here for file

Additional file 2 **R-Code**. This file contains the R-Code for the survival analysis. Click here for file
